# Specific and Efficient Uptake of Surfactant-Free Poly(Lactic Acid) Nanovaccine Vehicles by Mucosal Dendritic Cells in Adult Zebrafish after Bath Immersion

**DOI:** 10.3389/fimmu.2017.00190

**Published:** 2017-02-27

**Authors:** Julien Rességuier, Emilie Delaune, Anne-Line Coolen, Jean-Pierre Levraud, Pierre Boudinot, Dominique Le Guellec, Bernard Verrier

**Affiliations:** ^1^Laboratoire de Biologie Tissulaire et d’Ingénierie Thérapeutique, Université Claude Bernard Lyon 1, Centre National de la Recherche Scientifique (CNRS), Lyon, France; ^2^Macrophages et Développement de l’Immunité, Institut Pasteur, Centre National de la Recherche Scientifique (CNRS), Paris, France; ^3^Virologie et Immunologie Moléculaires, Institut National de la Recherche Agronomique (INRA), Université Paris-Saclay, Jouy-en-Josas, France

**Keywords:** poly(d,l-lactic acid) nanoparticles, surfactant free, zebrafish, vaccines carrier, mucosal delivery, dendritic cells, imaging flow cytometry, biodistribution

## Abstract

Activation of mucosal immunity is a key milestone for next-generation vaccine development. Biocompatible polymer-based nanoparticles (NPs) are promising vectors and adjuvants for mucosal vaccination. However, their *in vivo* uptake by mucosae and their biodistribution in antigen-presenting cells (APCs) need to be better understood to optimize mucosal nanovaccine designs. Here, we assessed if APCs are efficiently targeted in a spontaneous manner by surfactant-free poly(lactic acid) nanoparticles (PLA-NPs) after mucosal administration. Combining histology and flow imaging approaches, we describe and quantify the mucosal uptake of 200 nm PLA-NPs in adult zebrafish. Following bath administration, PLA-NPs penetrated and crossed epithelial barriers from all exposed mucosae. In mucosae, PLA-NPs accumulated in APCs, which were identified as dendritic cells (DCs), macrophages, and IgZ^+^ B cells in gills and skin. PLA-NP uptake by phagocytes was specific to these cell types, as PLA-NPs were not detected in neutrophils. Importantly, quantitative analyses in gills revealed that DCs take up PLA-NPs with specifically high efficiency. This study shows that surfactant-free PLA-NPs, which display optimal biocompatibility, can spontaneously target DCs with high efficiency *in vivo* following mucosal administration, and highlights PLA-NPs as powerful platforms for mucosal vaccine delivery in the medical and veterinary fields, and particularly in aquaculture.

## Introduction

Mucosae represent the first defensive barriers of the organism against most pathogens. Their defenses rely not only on physical barriers such as mucus production and mono- or multi-layered epithelia but also on specific immune systems called mucosa-associated lymphoid tissues (MALTs). The local activation of MALTs is currently a major challenge in vaccinology, both for human and veterinary medicine (1–3). In the fish farming industry, mucosal vaccines raise a growing interest compared to injected ones, as they offer wider possibilities for immunizing young fish, are easier to administer to large animal numbers, and cause less stress and tissue damage (4). As soluble antigens are poor mucosal immune inducers by themselves, extensive efforts have been made to develop particulate vaccine carriers able to cross the mucus and epithelial layers constituting mucosal barriers and deliver antigens to the key initiators of immune response, the antigen-presenting cells (APCs) (2, 3).

Some of the most promising vaccine vehicles for mucosal delivery are biodegradable polymeric nanoparticles (NPs), in particular NPs based on poly(d,l-lactic acid) (PLA) and its copolymer poly(lactic acid co-glycolic acid) (PLGA) (5, 6). PLA and PLGA are Food and Drug Administration-approved biocompatible lactate-based polyesters, which present high safety records, can be completely resorbed in the body by metabolization of their degradation products, are easy to produce and are eco-compatible (7). PLA- and PLGA-NPs constitute versatile vectors for adsorbing and/or encapsulating antigenic peptides and immunostimulant drugs (8, 9). They have been shown to induce immune responses against various model antigens following parenteral administration in mammals (2, 8). Their mucosal administration has also revealed their ability to cross mucosal barriers, reach some APCs, and elicit mucosal immune responses (6, 10–12). In fish, submicron PLGA particles have shown encouraging effects of immune response enhancement, following administration by injection and oral routes (9). However, a detailed understanding of *in vivo* uptake and biodistribution of polymeric NPs following mucosal administration is still lacking, which has limited their development as mucosal vaccine vehicles in vertebrates, and more particularly in fish. Here, we analyze if polymeric NPs efficiently cross fish mucosal barriers and reach APCs *in vivo*, using surfactant-free 200 nm poly(lactic acid) nanoparticles (PLA-NPs) as a model. PLA-NPs were prepared without any surfactant, for optimal biocompatibility (13). Because NPs generally become quickly coated by proteins and factors present in biological media (14), this model is likely representative of a variety of NPs which surface is functionalized using targeting or antigenic proteins.

We performed this study in adult zebrafish (*Danio rerio)*, a teleost fish model well suited for analyzing whole-body biodistribution with a cellular level of resolution, and benefiting from a rising number of available transgenic lines and molecular markers of immune cell types (15–17). The major lymphoid organs of teleosts are the kidney marrow, which is the site of hematopoiesis, the thymus and the spleen (18). Like other teleosts, zebrafish lack lymph nodes and display a diffuse lymphoid tissue organization in mucosae (4, 19). Zebrafish adaptive immune system becomes fully competent at juvenile stage (15, 20). They have three classes of immunoglobulins, M, D, and Z (called T in other teleosts), IgZ/T being the functional equivalent of mammalian mucosal IgA (21). Professional APCs such as macrophages, dendritic cells (DCs), and B cells have all been identified in zebrafish. Although it was in teleosts that the phagocytic ability of B cells was first recognized, it is still unclear if fish APCs fulfill similar roles to their mammalian counterparts in the initiation of adaptive immune responses (22, 23). Yet, DC cells capable of phagocytosis and T-cell stimulation were identified in zebrafish kidney marrow, and conserved co-stimulatory molecules (CD80/CD86 and CD83) have been detected in T-cell stimulating cells, further supporting a conservation of DC function in fish (24–26).

Main mucosae of adult teleosts are found in the gut, gills, skin, and nose; their MALTs are accordingly sub-divided into gut-, gill-, skin-, and nose-associated lymphoid tissues (GALT, GIALT, SALT, and NALT, respectively) (19, 21). Most leukocyte types are present in fish MALTs, including phagocytes (neutrophils, macrophages, DCs, and B cells), T cells, and plasma cells.

Here, we analyzed the biodistribution of PLA-NPs administered to adult zebrafish by immersion, a straightforward administration method targeting gut, gills, skin, and nasal mucosae altogether. We show that *in vivo*, surfactant-free PLA-NPs naturally penetrate and cross the mucosal epithelial barriers with high efficiency and are specifically accumulated in mucosal APCs, and not in neutrophils. Quantification in the gills reveals that DCs are the major APC type taking up PLA-NPs, with up to 65% being detected as positive for NPs. Our results suggest that active targeting strategies are not necessarily required for efficient uptake of polymeric NPs by mucosal DCs, and further highlight the potential of PLGA- and PLA-NPs as mucosal vaccine vehicles for applications in aquaculture.

## Materials and Methods

### NP Preparation and Characterization

Poly(lactic acid) nanoparticles were produced as previously described (10, 27). The red CellTrace BODIPY TR Methyl Ester (™) or green BODIPY 500/510 C4, C9 (™) fluorophores (Life Technologies) were encapsulated using a fluorophore:PLA ratio of 0.04% w/w. The NP size and size polydispersity were determined by diffuse light scattering using a Zetasizer nanoS apparatus (Malvern Instrument, UK).

### Fish Stocks, NP Administration, and Ethic Statement

Experiments were performed on wild-type AB/Tubingen zebrafish and transgenic *Tg(mhc2dab:GFP)sd6* (25), *Tg(mpeg1:mCherry)gl23* (28), *Tg(mpx:GFP)i114* (29), and *Tg(fli1:GFP)y1* (30) lines. Adults were individually immersed for 24 h at 28°C in 100 mL of fresh fish facility water, containing 0.01 or 0.05% fluorescent PLA-NPs. The experiments were conducted in accordance with the animal care guidelines of the European Union and French law, and the protocols were approved by the local Animal Ethic Evaluation Committee (No. CE015: Comité d’Evaluation Commun au Centre Léon Bérard, à l’Animalerie de transit de l’ENS, au PBES et au laboratoire P4—CECCAPP).

### Histology

Animals exposed to red NPs were euthanized by tricaine overdose, fixed in 4% PFA for 24 h at 4°C, then immersed in 30% sucrose for several days, embedded in Tissue-Tek O.C.T. Compound (Sakura Finetek USA), flash frozen in isopentane, and sectioned using a CM3050 S cryostat (Leica). DCs were stained using 1:50 FITC conjugate peanut agglutinin (FITC-PNA) (US Biological). Macrophage, neutrophil, and IgZ^+^ were stained using 1:250 rabbit anti-Mpeg1, 1:50 rabbit anti-Mpx, and 1:500 rabbit anti-IgZ-IN2 antibodies (AnaSpec), respectively, and 1:250 cross-adsorbed goat anti-rabbit secondary antibody (Thermo Fisher), which were either conjugated to DyLight 488 (for IgZ staining) or to DyLight 633 (for Mpx and Mpeg1 stainings). For double staining, cryosections were saturated with 5% BSA between FITC-PNA and antibody labeling. No cross staining was observed for PNA/IgZ labeling, while a weak PNA signal could be detected in a few Mpeg1^+^ macrophages. Mpx^+^ neutrophils consistently displayed moderate PNA signal, but distinct from DCs, which displayed high granular intracytoplasmic PNA staining and were Mpx^−^. Cryosections were co-stained with DyLight 488-Phalloidin (Thermo Fisher) and DAPI (Euromedex) and analyzed using a SP5 upward confocal microscope (Leica) with 63×/1.4NA objective and ImageJ.

### Flow Cytometry

The organs from euthanized animals (exposed to red PLA-NPs) were collected in cold PBS/heparin (1 U/mL)/FBS (2%). Cell suspensions from brain, gill, liver, spleen, and kidney were directly homogenized by passing through a 40-μm mesh filter (Fisherbrand). Skin and gut samples were dissociated for 8 min in 0.2% porcine trypsin (Sigma-Aldrich) in Versene solution (Life Technologies) before mesh filtration. Washed cell suspensions were treated with DAPI (2.5 μg/mL) to mark dead cells and processed using a LSRII Flow Cytometer (BD Biosciences). Data were analyzed using FlowJo v7.6.5.

### Imaging Flow Cytometry for Internalization Score

Cell suspensions, prepared as described above, were stained with 1:500 CellMask Green Plasma Membrane Stain (Life Technologies), treated with DAPI (10 μg/mL), and analyzed using an ImageStream^X^ Mark II imaging flow cytometer (Amnis, Millipore) with 63× objective, and IDEAs software. Cells with NP signal peaking at least sevenfolds over background were selected, and their cytoplasmic area (excluding membrane) was automatically determined based on CellMask signal. The internalization score, reflecting the ratio of cytoplasmic to total brightness intensity, was computed for each cell using a built-in IDEAS function. As a negative control for NP internalization, cell suspensions from unexposed fish were incubated with 0.002% red NPs for 30 min at 4°C.

### Imaging Flow Cytometry for Quantification of NP Uptake

For macrophages and neutrophils identification, cell suspensions were prepared from gills of *Tg(mpeg1:mCherry)gl23* or *Tg(mpx:GFP)i114* adults exposed to 0.05% green or red NPs (respectively); dead cells were marked using DAPI. For DC and IgZ^+^ cell staining, cell suspensions were prepared from gills of wild-type zebrafish exposed to red NPs, marked using 1:1,000 LIVE/DEAD Fixable Violet Dead Cell Stain Kit (Thermo Fisher) and fixed/permeabilized using 2% PFA/0.1% triton X-100. DCs were stained using 1:2,000 FITC-PNA in absence of FBS. IgZ^+^ cells were stained using 1:2,000 rabbit anti-IgZ (AnaSpec) and 1:2,000 DyLight488 conjugate cross-adsorbed goat anti-rabbit antibodies (Thermo Fisher). Samples were analyzed using ImageStream^X^ (50,000–100,000 events per acquisition) and IDEAS software. For DCs gating, events displaying the highest level of PNA staining were selected, from which cells devoid of an intracellular PNA signal or presenting a thick capsid (a hallmark of rodlet cells) were excluded.

### Statistical Analysis

Statistical analyses were performed using GraphPad Prism 6. Normality of samples was tested using Agostino and Pearson omnibus normality test. For flow cytometry experiments, one-way ANOVA coupled with Bonferroni multiple comparison *post hoc* analysis was carried on populations that passed the normality test (gills, skin, and kidneys). On populations that failed the normality assay (liver), the non-parametric equivalent Kruskal–Wallis test with Dunn’s multiple comparison *post hoc* analysis was used. For flow imaging experiments, as none of the populations respected the normality, a one-sided Mann–Whitney test was performed. Significance level is indicated as *p < 0.05, **p < 0.01, ***p < 0.001, ****p < 0.0001.

## Results

### PLA-NP Characterization

Poly(lactic acid) nanoparticles were produced by nanoprecipitation, a single step process whereby acetone-dissolved PLA is precipitated into a swirling aqueous phase of water and ethanol, in absence of surfactant. To detect PLA-NP by fluorescence imaging, red or green fluorescent dyes were encapsulated in NPs, by mixing the dyes with acetone. For optimal NP biocompatibility, water and acetone solvents were evaporated in the final product to a residual concentration well below 5,000 ppm, in accordance with European pharmacopeia standards. Red and green PLA-NPs displayed mean diameters of 225 and 200 nm, respectively, and were highly homogeneous in size within a batch, as shown by scanning electron microscopy and by diffuse light scattering indicating a low polydispersity index (Table 1; Figure S1 in Supplementary Material). Fluorophore-loaded PLA-NPs had a typical zeta potential of −60 mV.

**Table 1:** **Nanoparticle (NP) characteristics**.

NP	Fluorophore	Mean diameter (nm)	Polydispersity index
Red fluorescent—poly(d,l-lactic acid) (PLA)	CellTrace BODIPY TR methyl ester	225.4 ± 4.6	0.04 ± 0.01
Green fluorescent—PLA	BODIPY 500/510 C4, C9	199.9 ± 2.5	0.05 ± 0.01

### PLA NPs Are Taken Up by Epithelial Cells of All Targeted Mucosae

To study the mucosal uptake of PLA-NPs *in vivo*, we immerged zebrafish for 24 h in a bath containing 0.01% red NPs. This administration method targets gills, skin, nasal mucosa, and gut altogether and involves minimal manipulation that could be stressful to the fish or damaging to the tissues; indeed, the treatment did not cause more detectable stress than the transient increase of opercular breathing rhythm typically caused by netting and transfer of the fish from one tank to another. To determine PLA-NP biodistribution at whole-body level, NP localization was first analyzed by histological approaches, using confocal imaging of 40-μm-thick longitudinal whole-body cryosections. Images were taken within the core of cryosections, which allowed us to avoid the top and bottom edges of cryosections where we observed artifactual displacement of PLA-NPs related to cryosectioning and slide immersion (Figure S2 in Supplementary Material).

**Figure 1:**
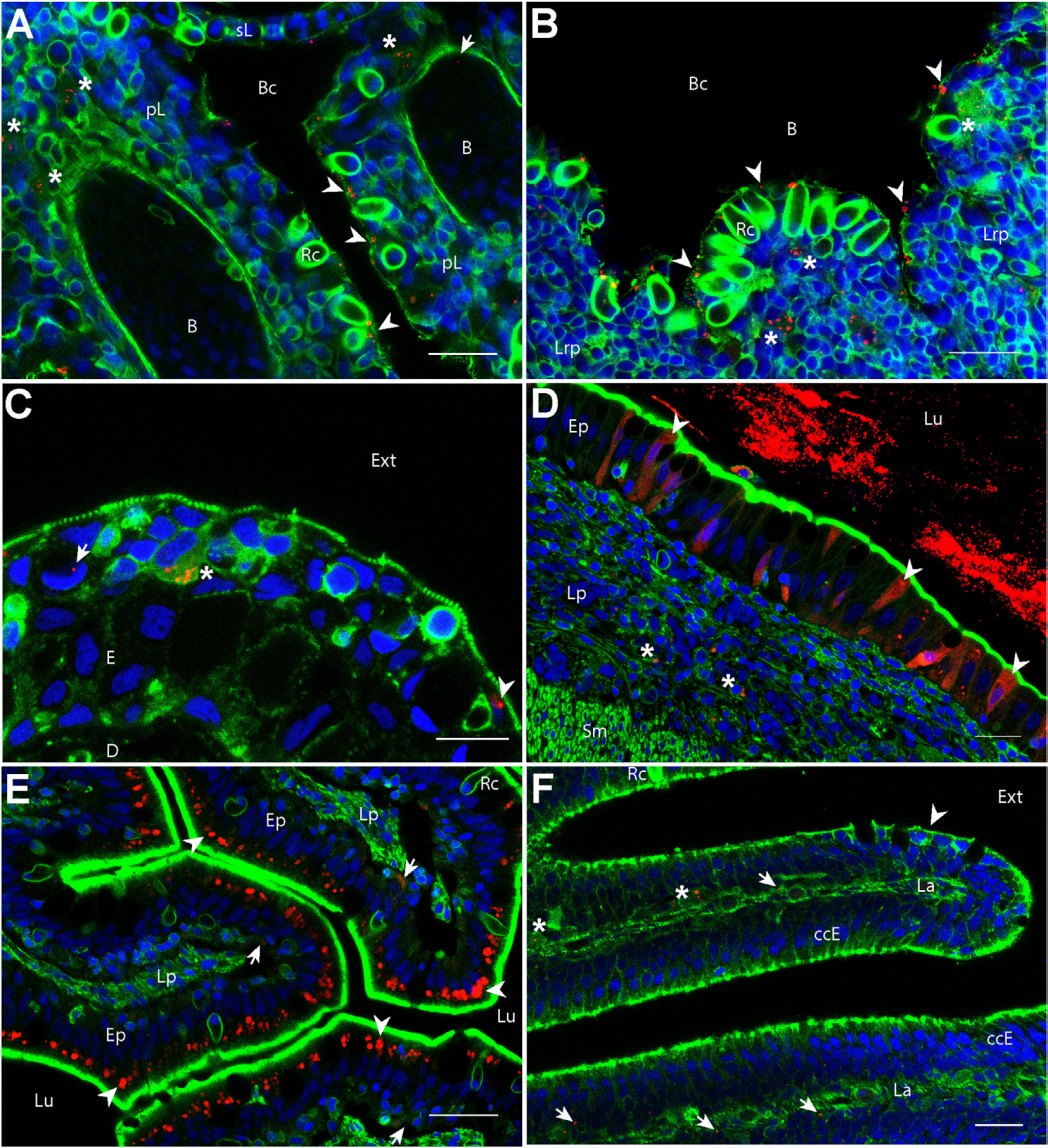
**Biodistribution of poly(lactic acid) nanoparticles (PLA-NPs) in mucosae and thymus**. Representative confocal images of gills **(A)**, thymus **(B)**, skin **(C)**, intestines **(D,E)**, and the olfactory mucosa **(F)**, of wild-type adults immersed for 24 h in 0.01% red fluorescent nanoparticles (NPs). Images were acquired in 40-μm-thick whole-body cryosections, stained with phalloidin (green) and DAPI (blue). **(A)** In gills, NPs are found in pavement cells lining the branchial cavity (arrowheads), accumulated in cells located in the sub-epithelial compartment (stars), and detected in the blood (arrow). **(B)** NPs are located in pavement cells lining the branchial cavity above the thymus (arrowheads) and accumulated in cells of the lympho-reticular parenchyma in the thymus (stars). **(C)** In skin, NPs are visible in keratinocytes (arrowhead), goblet cells (characterized by kidney-shaped nucleus, arrow) and accumulated in intra-epithelial cells (star). **(D,E)** In the intestine, a high number of NPs is found inside the intestinal *lumen* **(D)**. As illustrated in the end-gut **(D)**, NPs are taken up by enterocytes (arrowheads), which display a diffuse signal. A number of NPs is also observed in sub-epithelial cells (stars). In the post-midgut **(E)**, NPs are mainly found in apical areas of specialized enterocytes (arrowheads); a subset is also found at the basal epithelial side (arrows). **(F)** In the nasal mucosa, NP-positive cells are found in the olfactory epithelium (arrowhead) and the *lamina* (arrows). NPs are accumulated in large cells of the *lamina* (stars). B, blood vessels; pL, primary lamellae; sL, secondary lamellae; Bc, branchial cavity; Rc, rodlet cells; E, epidermis; D, dermis; Ext, external environment; Lu, *Lumen*; Ep, epithelium; Lp, *lamina propria*; Sm, smooth muscles; Lrp, lympho-reticular parenchyma; La, *lamina*. Scale bar: 10 μm **(B,E)**, 20 μm **(A,C,F)**, and 30 μm **(D)**.

We first investigated if PLA-NPs were taken up by epithelial cells of mucosae primarily exposed to NPs. In all these tissues, NPs were found in epithelial cells bordering the external environment (Figure 1). The gills, which have a gas-exchange function, typically display an extremely branched architecture of primary lamellae supporting secondary lamellae, which each consist in a network of capillaries sustained by endothelial pillar cells and covered by a non-keratinized squamous epithelium. PLA-NPs were detected in the epithelium lining the branchial cavity, and more specifically in pavements cells, identified by their typical actin microridges (Figure 1A). Located just below the gill cavity epithelium, the fish thymus may also be primarily exposed to NPs upon immersion administration (31). Indeed, we detected NPs in the outermost layer of pharyngeal epithelial cells covering the thymus (Figure 1B). The fish skin, unlike in mammals, is considered a mucosa: its outermost layer is made of living and proliferating cells coated with mucus and mucosal IgZ/T antibodies (32). The epithelial cells form a stratified non-keratinized epidermis, interspersed with pores located over various types of mucus-secretory cells. We detected PLA-NPs both in keratinocytes and in goblet cells (Figure 1C). As in mammals, the fish gut displays a single epithelial layer mainly composed of brush-bordered enterocytes and mucus-producing goblet cells, supported by folds of connective *lamina propria* (33). We detected PLA-NPs in enterocytes throughout the intestine (Figures 1D–E), with a particularity in the post-midgut, where a high concentration of NPs was observed in supranuclear vacuoles of so-called specialized enterocytes, which were described as a functional equivalent of the antigen-sampling mammalian M-cells (34, 35) (Figure 1E). NPs were also observed in the nasal mucosa, in cells of the ciliated pseudostratified columnar epithelium (Figure 1F). Thus, despite the mucus layer covering mucosal epithelia, PLA-NPs reached and were taken up by epithelial cells of all exposed mucosae.

### PLA NPs Cross Mucosal Epithelial Barriers and Reach Mucosal APCs

We then analyzed if PLA-NPs were able to cross epithelial barriers to reach mucosal APCs, in cryosections. We detected NP accumulations in cells located in the deeper layers of the skin and the gills, in the *lamina propria* of the gut, in the lympho-reticular parenchyma of the thymus and in the nasal *lamina* (Figure 1). In the post-midgut, NPs were also found translocated at the basal boundaries of specialized enterocytes (Figure 1D). A variety of leukocytes, including phagocytes, are present in deep epithelial and sub-epithelial areas (19). The pattern of NP accumulations suggested that these NP-positive cells could be phagocytes.

**Figure 2:**
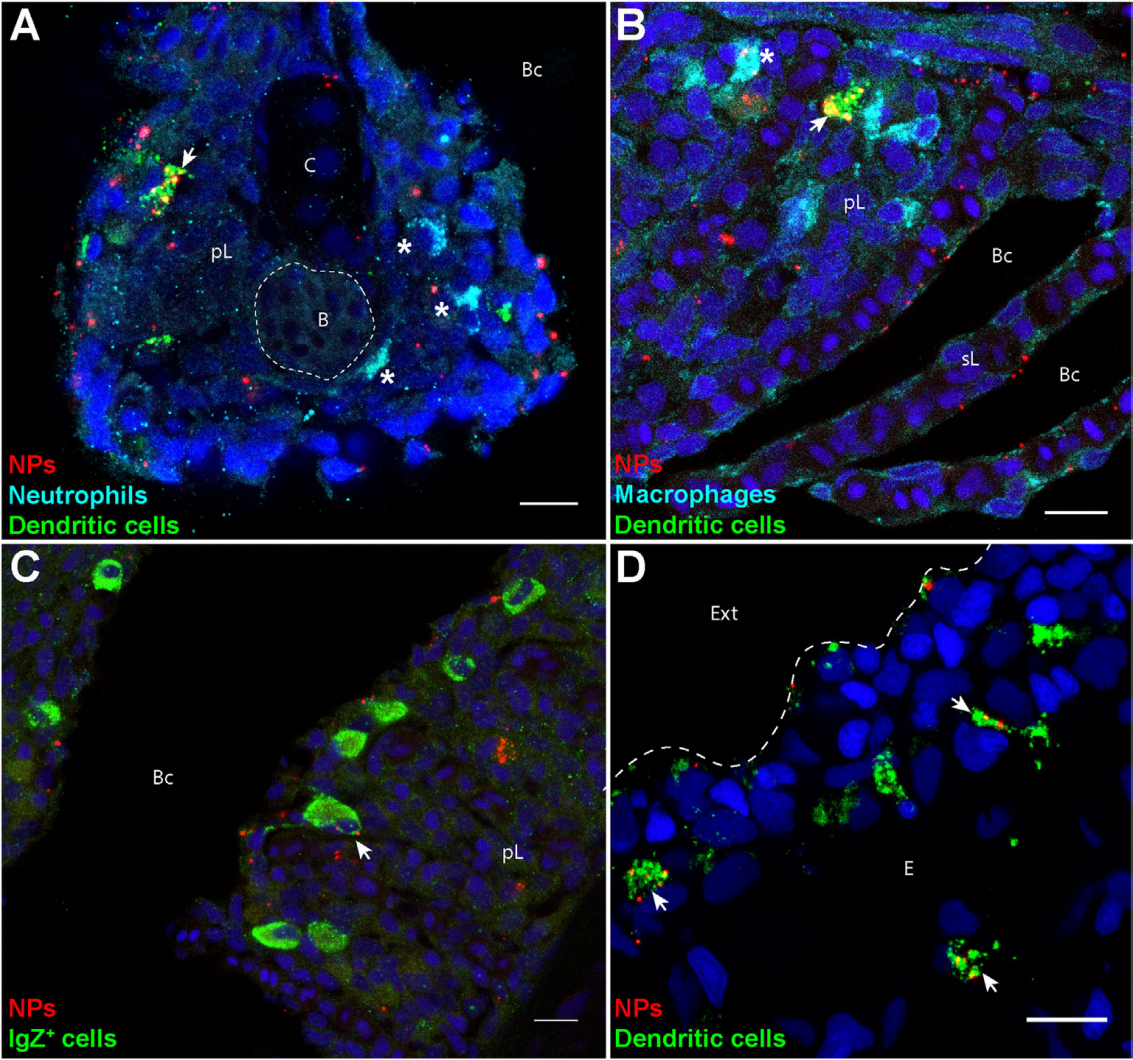
**Poly(lactic acid) nanoparticles (PLA-NPs) are accumulated in mucosal antigen-presenting cells**. **(A–D)** Representative confocal images of gills **(A–C)** and skin **(D)** of wild-type adults immersed for 24 h in 0.01% fluorescent PLA-NPs (red). Images were acquired from 40-μm-thick whole-body cryosections, stained for neutrophils (Mpx-positive, cyan) **(A)**, macrophages (Mpeg1-positive, cyan) **(B)**, IgZ^+^ B-cells (green) **(C)**, and dendritic cells (DCs) (PNA, green) **(A,B,D)**, in addition to nuclei (DAPI, blue). Maximal intensity projections from 3 **(A–C)** or 5 **(D)** optic sections acquired every micrometer. Nanoparticle (NP) accumulations are observed in gill DCs [**(A,B)**, arrows], macrophages [**(B)**, star], and IgZ^+^ cells [**(C)**, arrow], while no NP are detected in neutrophils [**(A)**, stars]. NPs are also taken up by a network of skin DCs [**(D)**, arrows]. pL, primary lamellae; sL, secondary lamellae; Bc, branchial cavity; B, blood; C, cartilage; Ext, external environment; E, epidermis. Scale bar: 10 μm.

To identify these cells, we labeled gills and skin cryosections using specific markers, such as Mpx and Mpeg1, expressed in neutrophils and macrophages, respectively (28, 29). IgZ staining was used to identify cells of the B-cell lineage specialized in mucosae protection, as described in trout (36). The identification of DCs was performed based on their affinity for PNA, a surface marker of live DCs (24). In fixed, permeabilized cryosections, cells with the highest PNA^+^ signal exhibited a granular intracytoplasmic staining and cellular protrusions reminiscent of dendrites. They displayed a strong MHC class 2 staining in transgenic *mhc2dab:EGFP* fish (Figure S3 in Supplementary Material) and were negative for Mpeg1 and IgZ stainings (Figure 2), further suggesting that these are DCs.

Following PLA-NP exposure, DCs displayed a strong uptake of NPs in gills (Figures 2A,B), as well as in skin (Figure 2D). In gills, PLA-NPs were also observed in a number of macrophages (Figure 2B) and less frequently in IgZ^+^ cells (Figure 2C). Interestingly, no NPs were detected in neutrophils (Figure 2A).

### PLA NPs Enter the Bloodstream

**Figure 3:**
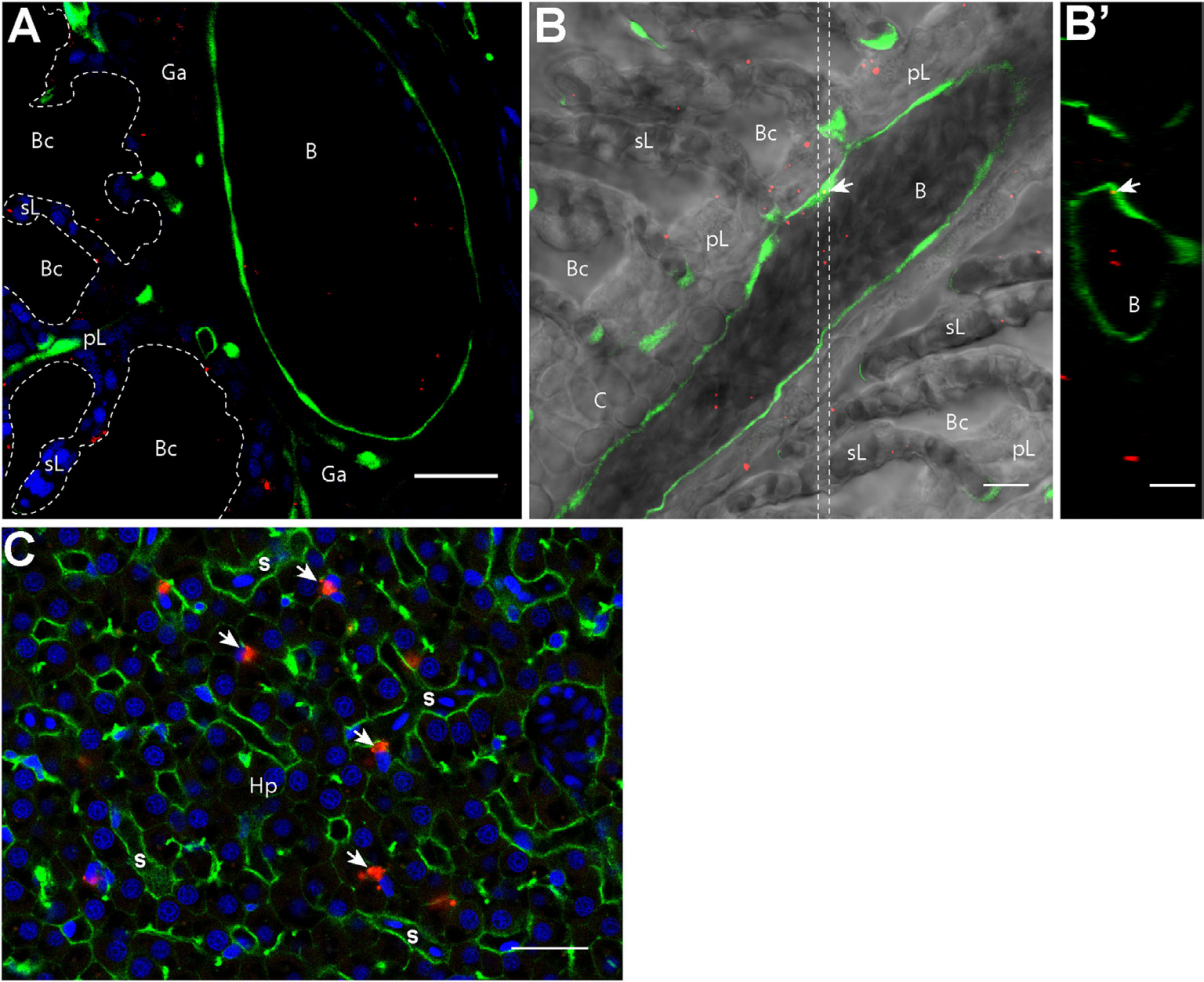
**Nanoparticles (NPs) reach the circulatory system and the liver**. **(A,B′)** Representative confocal images of gills from transgenic fli:GFP adults bathed for 24 h in 0.05% red fluorescent NPs. Images were acquired in 40-μm-thick whole-body cryosections, stained with DAPI [blue in **(A)**]. NPs are found in the *lumen* of gill arch **(A)** and gill filament **(B)** blood vessels, which are delimited by green endothelial cells. NPs were also detected inside endothelial cells [arrow in **(B)**]. **(B′)** These observations are confirmed by the orthogonal view realized in the area between the two dotted lines in **(B)**. **(C)** Representative confocal image of the liver of wild-type adults exposed to 0.01% red fluorescent NPs. Images were acquired in 40-μm-thick whole-body cryosections, stained with phalloidin (green) and DAPI (blue). NPs are highly concentrated in cells close to sinusoids (s) and displaying an oblong nucleus (arrows). pL, primary lamellae; sL, secondary lamellae; Bc, branchial cavity; B, blood vessel; Ga, gill arch; C, cartilage; Hp, hepatocytes. Scale bar: 10 μm **(B,B′)**, 20 μm **(A,C)**.

In addition to their accumulation in APCs, we detected scattered PLA-NPs in the circulatory system, as seen in blood vessels of the primary lamellae in gills (Figure 1A). Immersion exposure of transgenic *fli:GFP* adult zebrafish to fluorescent NPs at higher concentrations (0.05%) revealed not only numerous PLA-NPs in the gill bloodstream (Figure 3) but also NP internalization in endothelial cells (Figures 3B,B’). Thus, PLA-NPs cross mucosae and enter the bloodstream, suggesting that NPs may also reach internal organs.

Indeed, NP accumulations were observed in liver cells close to sinusoids (Figure 3C). These cells, which displayed an oblong nucleus distinct from the round shaped nucleus with scattered chromatin of hepatocytes, may correspond to macrophages or hepatic stellate (Ito) cells (37, 38). NPs were never detected in brain, spleen, or kidney cryosections.

### PLA NPs Penetrate the Organism in a Dose-Dependent Manner and Reach Liver and Kidney

**Figure 4:**
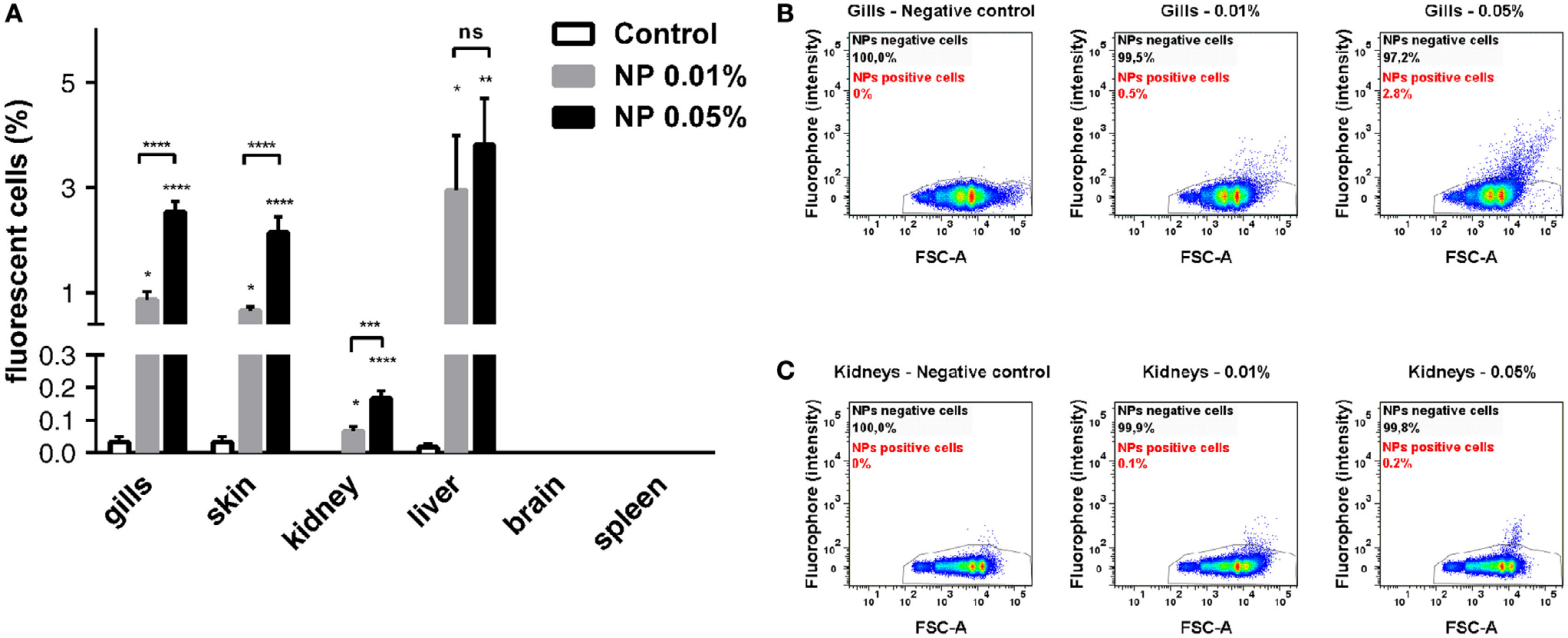
**Quantitative analysis of poly(lactic acid) nanoparticles (PLA-NPs) uptake in primary and secondary exposure organs**. **(A)** Flow cytometry analysis of nanoparticle (NP) uptake in gills, skin, kidney, liver, brain, and spleen, following exposure to 0.01 or 0.05% red fluorescent NPs. Histograms represent the mean percentage of positive cells for each organ. While no NP uptake was detected in brain and spleen, PLA-NPs significantly penetrated all other organs in a dose-dependent manner, except for the liver where no significative fluctuation was observed. **(B,C)** Representative biparametric representations of NP uptake in gills **(B)** and whole-kidney marrow **(C)**. NP-positive cells are mainly found among FSC-high cells. Error bars: SEM. Significance level is indicated as: *p < 0.05, **p < 0.01, ***p < 0.001, ****p < 0.0001. Number of fish per condition (control/0.01/0.05%): gills (9/13/9), skin (9/13/9), kidneys (12/12/12), liver (12/12/12), brain (6/6/6), and spleen (6/6/6).

To quantify the PLA-NP uptake in primary and secondary exposure organs, we performed flow cytometry on dissociated cells of various candidate organs, which were dissected from fish exposed for 24 h to 0.01 or 0.05% fluorescent NPs by immersion (Figures 4A–C). Consistent with confocal observations, gills and skin displayed 0.86 and 0.65% NP-positive cells, respectively, following 0.01% NP exposure. A dose-dependent uptake was observed in these organs, among which the proportion of NP-positive cells tripled (2.5 and 2.1%, respectively), for 0.05% NP exposure. Quantification of NP uptake by this approach was, however, not applicable to intestines, due to the large number of debris corresponding to mucus and intestinal content loaded with NPs (not shown).

Regarding internal organs, flow cytometry analysis corroborated the absence of NP uptake in the brain and the spleen observed by histology approaches. In kidney, however, a small population of NP-positive cells was detected by flow cytometry, increasing with NP concentration, but reaching only 0.16% at the highest NP concentration (Figure 4C). Consistent with histological observations, a higher mean of 3.5% positive cells was detected by flow cytometry in the liver for 0.01% NP exposure. Contrary to other organs, the proportion of liver NP-positive cells showed no significant difference between the two PLA-NP concentrations, suggesting that liver capacity may be reached at low NP concentration, as least in term of cell number.

### PLA NPs Are Actively Internalized and Accumulate in Kidney Cells with Myeloid Cell Features

**Figure 5:**
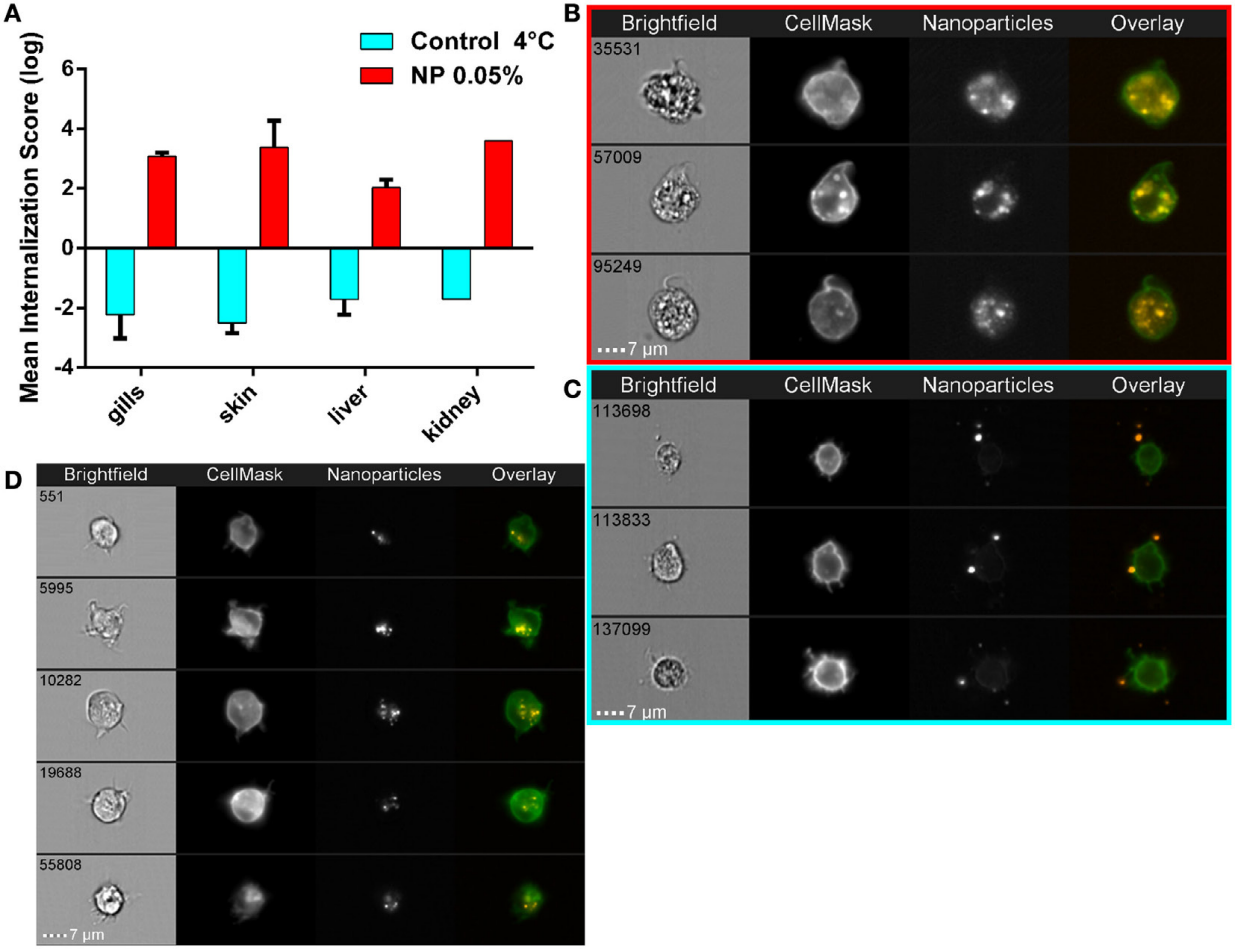
**Active nanoparticles (NPs) internalization in cells of mucosae, kidneys, and liver**. **(A)** NP internalization score, assessed by imaging in flow, in dissected organs from adult zebrafish exposed to 0.05% red fluorescent NPs (red histograms). As a negative control for internalization (blue histograms), PLA-NPs were added at 4°C just before acquisition on the cell suspension from fish unexposed to NP. Whereas most NPs are found at or outside the cell membrane in the negative controls (internalization scores <−1), a high majority of NPs is detected inside the cells of NP-treated fish (internalization scores >2) in all analyzed organs. **(B**–**D)** Representative images of NPs in kidney **(B,C)** or gill **(D)** cell suspensions, from NP-exposed fish **(B,D)** or control conditions **(C)**, which display membrane processes reminiscent of dendrites. Total number of analyzed cells (NP 0.05%/4°C control): gills (three independent acquisitions) 1312/4355, skin (two independent acquisitions) 345/4185, liver (two independent acquisitions) 1431/2651, and kidney (one acquisition) 117/3180. One fish per acquisition. Error bars: SD.

Because flow cytometry does not allow discrimination of true NP internalization events from cases where NPs are simply stuck on the plasma membrane, we performed imaging flow cytometry to clarify this point. Single cells isolated from fish exposed to 0.05% PLA-NPs displayed a clear intracellular NP localization, as revealed by the use of a plasma membrane marker, and illustrated in kidney cell suspensions (Figures 5A,B). The signal pattern was different when cells were exposed *in vitro* to PLA-NPs under conditions that block internalization (4°C), where NPs were seen aside cells or stuck on their plasma membrane (Figure 5C). This was quantified by computing internalization scores based on a large number of single cells (Figure 5A). Altogether, PLA-NP internalization score was 1,000 times higher for cells of NP-exposed fish, than for the 4°C control. These data not only validate the consistency of our previous flow cytometry results but also reveal that PLA-NPs are actively taken up in cells of primary and secondary organs, following fish immersion.

Interestingly, as for the large gill cells with NP accumulations, the NP-positive kidney cells displayed cellular processes reminiscent of dendrites (Figures 5B,D), suggesting that these might be myeloid cells and possibly DCs.

### DCs Are the Major Mucosal APCs Taking Up NPs *In Vivo*

The spontaneous targeting of PLA-NPs to APCs after mucosal administration (Figure 2) unveiled their promising potential as mucosal vaccine vectors. To quantify in a robust manner their tropism for phagocytes, we used high-resolution flow microscopy on single cell suspensions from fish exposed for 24 h to 0.05% fluorescent NPs. This was performed on gills, which are not only easy to dissociate, but also represent an organ of high significance for host-pathogen interactions, offering a wide area of contact with the environment (39). In these experiments, specific labeling of neutrophils and macrophages was achieved using *mpx:eGFP* and *mpeg1:mCherry* transgenic fish, respectively, while DCs and IgZ^+^ cells were identified, based on previous histological results (Figure 2), as cells with strong granular and intracellular PNA staining, and cells with strong IgZ signal, respectively.

**Figure 6:**
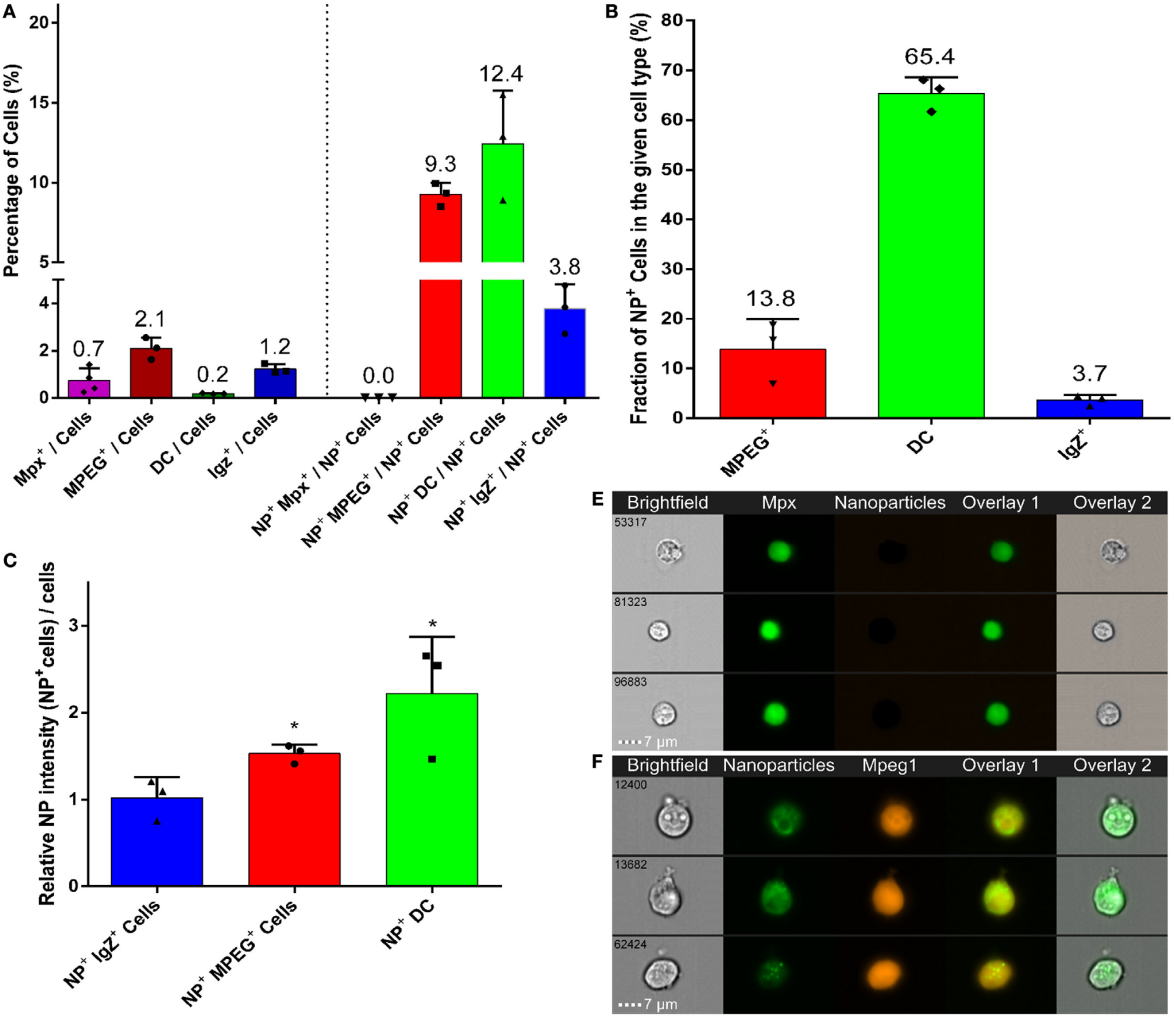

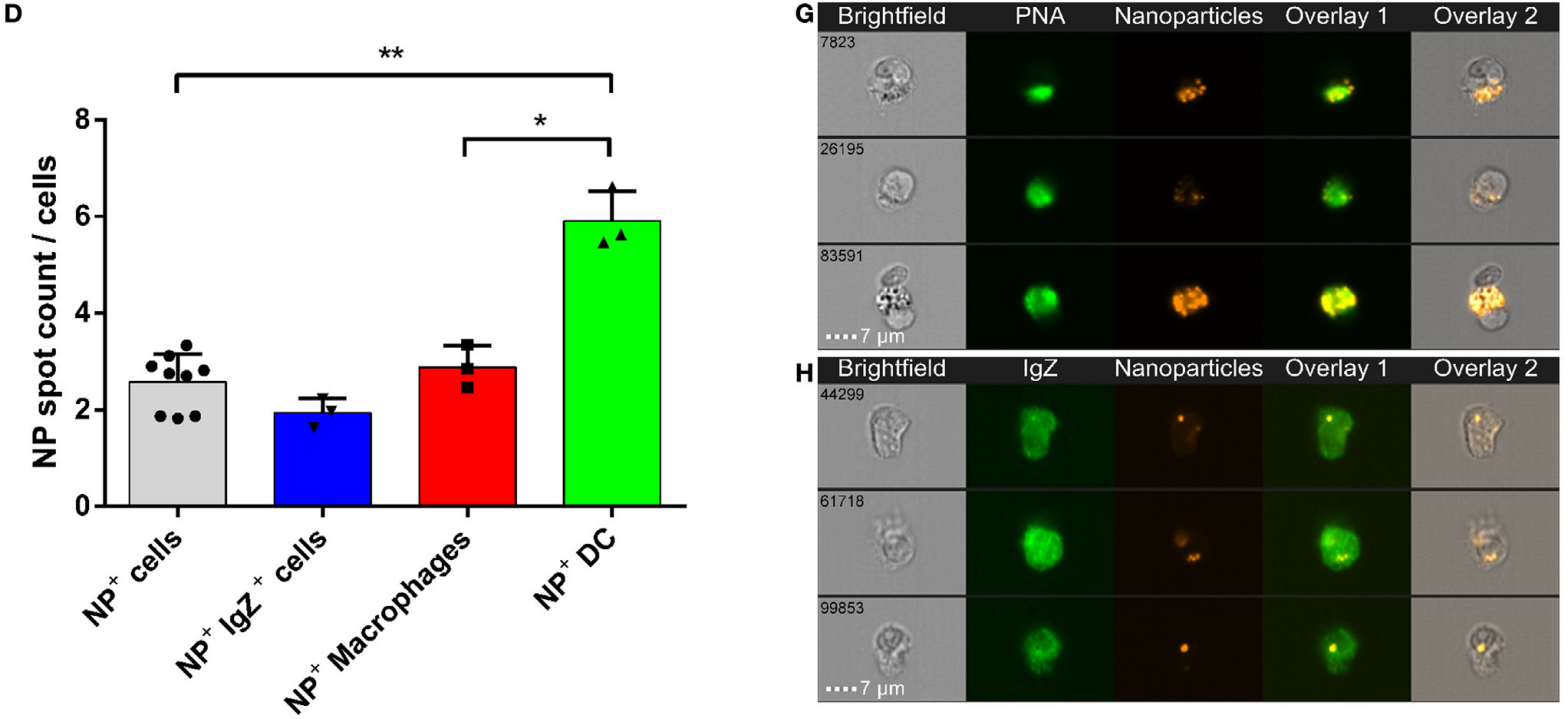
**High uptake of poly(lactic acid) nanoparticles (PLA-NPs) by mucosal dendritic cells (DCs)**. **(A–D)** Quantification by imaging flow cytometry of the nanoparticle (NP) uptake by gill phagocytes from adult zebrafish, previously immersed for 24 h in 0.05% NPs. For each cell type, results from at least three independent acquisitions are represented as dots; histograms indicate the mean. Neutrophils and macrophages were labeled by GFP and mCherry expression in transgenic *mpx:GFP* and *mpeg1:mCherry* fish, respectively (one fish per acquisition). DCs and IgZ^+^ B-cells were identified based on high intracellular and granular FITC-PNA staining and intracellular IgZ staining, respectively (two fishes per acquisition). **(A)** Percentage of NP-positive cells, for each phagocyte type, relative to the total number of cells (left) or of NP-positive cells (right). APCs, which represent less than 4% of total cells in gills, represent over 25% of NP-positive cells altogether, among which DCs show the most important enrichment. **(B)** Percentage of NP-positive cells within each cell type. A high majority of DCs are positive to PLA-NPs. **(C)** NP signal intensity per cell relative to the mean fluorescence of all NP-positive cells. DCs and macrophages internalize more NPs than average. **(D)** NP signal spot count per cell. DCs display more signal *foci* than average. **(E**–**H)** Representative images of neutrophils **(E)**, NP-positive macrophages **(F)**, DCs **(G)**, and IgZ^+^ cells **(H)**. Error bars: SD. Significance level is indicated as: *p < 0.05, **p < 0.01.

Phagocytes represented over 25% of all NP-positive cells, although they constitute less than 5% of total gill cells (Figure 6A). Of these, DCs were the most abundant NP-positive phagocytes (12.4% of total NP-positive cells) (Figures 6A,G). Macrophages and IgZ^+^ cells represented 9.3 and 3.8% of total NP-positive cells, respectively (Figures 6A,F,H). Moreover, we confirmed the absence of NP detection in neutrophils (Figures 6A,E). The 60-fold enrichment in DC proportion in NP-positive cells, compared to their overall proportion among total gill cells, is especially striking. This ratio is to be compared to the fivefold enrichment for macrophages and threefold enrichment for IgZ^+^ cells. This strong uptake tendency of DCs was additionally evidenced by NPs being detected in 65% of all DCs (Figure 6B). By contrast, PLA-NPs were detected in only 13.8 and 3.7% of macrophages and IgZ^+^ cells, respectively.

Poly(lactic acid) nanoparticles were not only taken up by many DCs but also present in large amounts in these cells. Indeed, relative to the average intensity detected for all NP-positive cells, DCs and macrophages, but not IgZ^+^ cells, displayed significantly higher signal (Figure 6C). When a “spot count” analysis was applied, DCs significantly displayed the highest number of intracellular PLA-NP spots (on average 5.9 spots/cell), compared to macrophages (on average 2.1 spots/cell) and IgZ^+^ cells (on average 1.9 spots/cell) (Figure 6D).

Altogether, our results demonstrate that following mucosal administration, surfactant-free PLA-NPs are not only spontaneously internalized by epithelia but also efficiently accumulated in mucosal APCs and especially in DCs, which likely represent crucial target APCs of vaccine approaches.

## Discussion

An increasing body of evidence has revealed the potential of biodegradable polymeric NPs for mucosal immunization, in the medical and veterinary fields, and in particular in aquaculture, where oral, dipping, and balneation methods of administration are more convenient and less damaging than injection. Yet, designing strategies to further enhance efficacy of polymeric NPs as mucosal vaccine vehicles requires a better understanding of how they are taken up by mucosae and get distributed in the organism, an aspect that has been often overlooked in the field at the expense of functional immunization studies. Here, using 200 nm PLA-NPs as model NPs administered to adult zebrafish, we analyzed if surfactant-free polymeric NPs are efficiently naturally taken up by mucosae and to what extent they reach DCs, which in mammals are key professional APCs leading the immune response. Zebrafish were immersed for 24 h in a bath of fluorophore-loaded PLA-NPs, a simple administration mode that altogether targets mucosae in gills, gut, skin, and nasal cavities. Using an unbiased histological approach at whole-body level, combined to quantification by flow cytometry and advanced flow imaging in dissected organs, we showed that PLA-NPs crossed mucosal barriers of all analyzed mucosae and accumulated with specifically high efficiency in mucosal APCs, in particular in DCs. As surfactant-free PLA-NPs are most likely quickly covered by factors present in immersion water and biological tissues (14), the biodistribution of non-functionalized PLA-NPs that we describe here may also be indicative of the general tropism of surfactant-free polymeric NPs, functionalized or not.

### Surfactant-Free PLA-NPs Efficiently Cross Mucosal Barriers

At the mucosal level, we observed a conserved pattern whereby PLA-NPs penetrated cells of the outer epithelial layer and accumulated in phagocytes of deeper layers (Figures 1 and 4). PLA-NPs were detected in pavement epithelial cells and phagocytes of gills, skin, and nose, in accordance with previous studies that examined the uptake of microspheres, bacteria, or nanoliposomes, in trout and zebrafish (40–43). PLA-NPs were also found in the epithelium and phagocytes of the nasal mucosa, which constitutes a promising site for mucosal vaccination in fish and mammals (44, 45). Furthermore, PLA-NPs swallowed during immersion (46) were very efficiently taken up by enterocytes, which displayed strong and diffuse NP signals in most gut regions, and were detected in phagocytes of the *lamina propria*, in agreement with other studies that addressed the intestinal uptake of bacteria, submicron polymeric NPs or proteins (34, 47). High amounts of PLA-NPs were also detected in supranuclear vacuoles of post-midgut specialized enterocytes. These cells were shown to take up foreign material such as proteins, gold NPs or bacteria from the gut *lumen*, and store it in supranuclear vacuoles, and were suggested to play an antigen-sampling role similar to mammalian M-cells (34, 35, 43, 47). Supporting this notion, we detected accumulations of PLA-NPs at the basal side of the specialized enterocyte epithelium (Figure 1D). Although we cannot rule out that PLA-NP sampling is directly performed in the *lumen* by phagocytes themselves, these observations confirm that highly efficient antigen uptake takes place in the post-midgut region.

Thus, PLA-NPs efficiently cross epithelial barriers of all exposed mucosae. The fate of NPs taken up by mucosal epithelial cells and phagocytes, and the path taken by NPs from mucosal surfaces to the blood vessels remain to be investigated, to determine to what extent phagocytes contribute to this transport and if other transport mechanisms occur in mucosae.

### Surfactant-Free PLA-NPs Reach a Subset of Organs with Immune-Related Functions

Being positioned in the gill cavities, the thymus, which is the primary lymphoid organ for T lymphocytes development (31), seems primarily exposed to foreign antigens. As in mucosae, PLA-NPs were detected in the outer layer of epithelial cells covering the thymus and accumulated in cells of deeper layers, suggesting an intriguing role of the thymus as antigen-sampling organ. This paradox, for an organ that plays a key role in self-non-self discrimination, deserves further study.

We furthermore demonstrated that PLA-NPs reach a subset of internal organs. The accumulation pattern of NPs in liver cells morphologically distinct from hepatocytes suggests that these cells may be macrophages or hepatic stellate cells, which in mammals not only have the ability to engulf NPs but were also described as liver-resident professional APCs able to induce potent immune responses (37, 38, 48). The possibility that liver NP-accumulating cells may be APCs remains to be explored. We also identified by flow cytometry the presence of a small amount of PLA-NP-positive cells in kidney, which in teleosts is the main primary lymphoid organ and a site for antigen presentation and lymphocyte maturation, as well as the niche for antibody producing cells (49). Imaging flow cytometry on isolated kidney cells revealed the presence of NP-accumulating cells with processes reminiscent of dendrites, suggesting that NPs might be present in DCs in this organ. The functional importance of NP presence in thymus, liver, and kidneys for immune response remains to be further investigated. In the spleen, confocal microscopy and flow cytometry did not reveal any PLA-NP signal above the high intrinsic fluorescence. While PLGA-NPs accumulate in the spleen shortly after being administered by gavage, polystyrene beads do not (47). Moreover, *Yersinia* antigens were detected in the spleen after immersion administration, but not 125 nm nanoliposomes (43, 50). Size difference, administration method, and NP composition may strongly influence particulate carrier uptake by the spleen.

The presence of PLA-NPs in spleen and kidney raises questions regarding the path taken by NPs to reach internal organs. Although we cannot exclude that PLA-NPs diffuse through the mesenchymal tissue, their detection in gill blood vessels strongly suggests that PLA-NPs were transported to liver and kidney by the bloodstream. PLA-NPs, which have a hydrodynamic diameter too large to undergo glomerular clearance (51), may travel freely in the bloodstream or may be transported by APCs.

Altogether, the ability of PLA-NPs to penetrate and accumulate in all mucosae and lymphoid organs, with the exception of the spleen, is promising for fish mucosal vaccine development. Moreover, the absence of PLA-NP detection in the brain by flow cytometry suggests that PLA-NPs were not able to cross the blood–brain barrier, reducing safety concerns for vaccine delivery.

### Surfactant-Free PLA-NPs Efficiently Target DCs and Other APCs

Poly(lactic acid) nanoparticles accumulations were observed in mucosal phagocytes, corresponding to the three professional classes of APCs: macrophages, B cells, and DCs. Surprisingly, we did not observe PLA-NP uptake in zebrafish neutrophils, which contrasts with reports of PLA-NP uptake by circulating neutrophils following intravascular injections in guinea pigs or by rat granulocytes *in vitro* (52, 53). This difference may be explained by variations in NP physicochemical characteristics, such as the amount of surfactant, as polyvinyl alcohol was used for the preparation of the PLA-NPs described in these reports (52, 53). Although we cannot exclude that PLA-NPs are taken up by neutrophils with a different kinetics than in other APCs, this hypothesis is unlikely, because fish exposure to NPs was continuous in this protocol. One may also speculate that neutrophils need to be activated (e.g., by local danger signals associated with the injection) to become able to engulf NPs, but they did not in zebrafish larvae injected with PLA-NPs (unpublished observations).

Instead, we detected NP accumulations in mucosal APCs, which, after 24 h of cumulative exposure to PLA-NPs, represented in gills 25% of all NP-positive cells. Of these NP-positive APCs, half were macrophages or B cells. The initial dogma that B cells were incapable of phagocytosis *in vivo* was broken by the finding that teleost B cells are able to engulf and kill pathogens (54). Our results showing that PLA-NPs are taken up by gill IgZ^+^ cells support the phagocytic nature of zebrafish B cells, in agreement with other studies (20, 25). The macrophages function as cleaners of the organism, performing rapid phagocytosis, and neutralization of encountered foreign materials and debris. Less potent than DCs, they are nevertheless also involved in immune response triggering. Given the great representation of macrophages and B cells in mucosae (2 and 1%, respectively, in gills), the ability of these APCs to take up PLA-NPs represents an interesting prospect for the use of particulate NPs in mucosal vaccines.

A major part of NP-positive mucosal APCs, which represented over 10% of all NP-positive cells in gills, were DCs characterized by the following features: high intracellular and granular PNA staining (in fixed/permealized tissues), dentritic-like morphology, MHC2 expression, and absence of both Mpeg1 and Mpx expression. Although we cannot dismiss that all DCs might not share these characteristics, DC quantification in gills was in accordance with the macrophage/dendritic cell ratio previously described (25). In gills, where DCs represent only a small population of total cells, over 65% of DCs were positive to PLA-NPs and displayed the highest PLA-NP accumulations of all APCs, showing the great efficiency of DCs for PLA-NP uptake. Having unique ability to efficiently prime naïve T cells, mammalian DCs play a key role in orchestrating the activation and modulation of the adaptive immune response. DCs thus represent a crucial target for the development of mucosal vaccines, and the high efficiency of PLA-NP uptake by mucosal DCs, in absence of targeting molecules, makes PLA-NPs vehicles of choice for mucosal vaccine delivery.

## Conclusion

Altogether, we show that, in absence of functionalization, surfactant-free polymeric NPs of 200 nm are able to cross mucous layers and mucosal epithelia, and to be efficiently taken up by DCs. Thus, balneation in polymeric NPs may represent a convenient and efficient way to deliver vaccine principles to batches of adult and young fish in the aquaculture industry. Although the teleost lymphoid tissues are diffuse, zebrafish mucosae share many structural and functional similarities with mammalian mucosae, suggesting that the efficient targeting of DCs by mucosally applied polymeric NPs may be general among vertebrates, with potential applications in human health. Further work will be needed to investigate the ability of antigen-carrying polymeric NPs to induce efficient immune responses by mucosal routes. For this, functionalization strategies designed for improving the targeting *per se* of NPs toward DCs may be less beneficial for vaccine efficiency than other strategies aimed at activating DCs. Altogether, thanks to their versatility for carrying immune-stimulating molecules and antigens, their biodegradability, their eco-compatibility, and, as we show here, their inherent APC targeting ability, polymeric NPs using PLA backbone, represent great prospects for the enhancement of mucosal vaccines, with potential application in human health and aquaculture.

## Author Contributions

JR participated in the conception and design of the study, performed experiments, analyzed data, and wrote the manuscript. ED and DG participated in the conception and design of the study, assisted in experiments and data analysis, and wrote the manuscript. A-LC performed histology in *fli:GFP* lines, analyzed data, and assisted in drafting the manuscript. J-PL generated *mhc2dab:eGFP* samples, assisted in designing the study, and helped revise the manuscript. PB assisted in designing the study and helped revise the manuscript. BV assisted in the conception and design of the study, revised the manuscript, and provided reagents and lab space. All the authors read, critiqued, and approved the final manuscript.

## Conflict of Interest Statement

BV is cofounder in a company developing the therapeutic use of PLA nanoparticles. The remaining authors declare no commercial or financial relationships that could be construed as a potential conflict of interest.
