# Supplementary material for: Specific and Efficient Uptake of Surfactant-Free Poly(Lactic Acid) Nanovaccine Vehicles by Mucosal Dendritic Cells in Adult Zebrafish after Bath Immersion
Source: Front Immunol. 2017 Feb 27;8:190. doi: 10.3389/fimmu.2017.00190 (PMC5326745; doi:10.3389/fimmu.2017.00190)
Supplement: Supplementary file 1 [file Data_Sheet_1.DOCX]

Supplementary Material

Specific and Efficient Uptake of Surfactant-Free Poly(Lactic Acid) Nanovaccine Vehicles by Mucosal Dendritic Cells in Adult Zebrafish after Bath Immersion

J Rességuier^a^, E Delaune^a^, AL Coolen^a^, JP Levraud^b^, P Boudinot^c^, D Le Guellec^a^, B Verrier^a^*

*** Correspondence:** Dr.Bernard VERRIER: [bernard.verrier@ibcp.fr](mailto:bernard.verrier@ibcp.fr)





**Supplementary Figure 1.** Scanning electron microscopy acquisition of 200 nm surfactant-free PLA nanoparticles

**
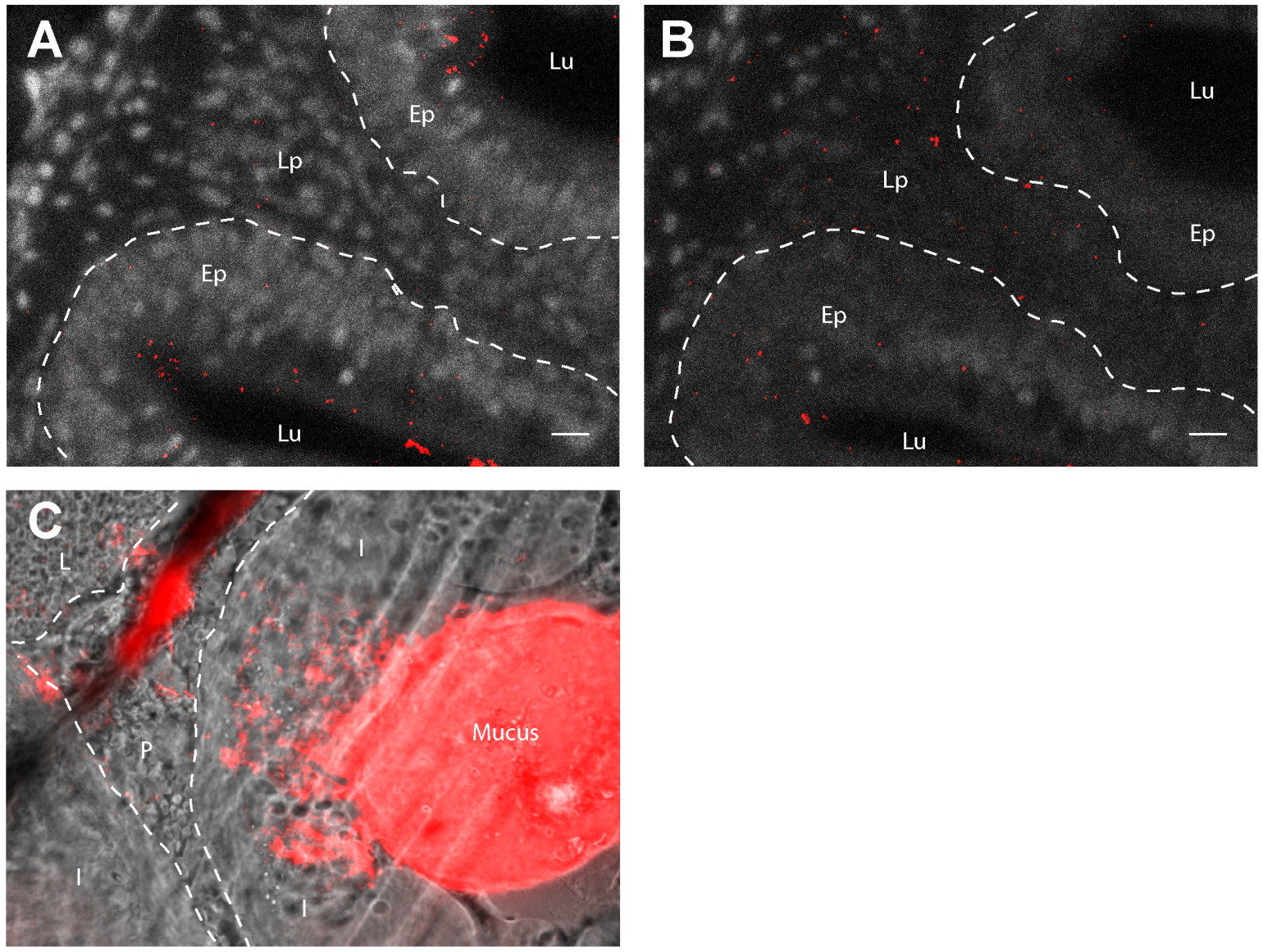
**

**Supplementary Figure 2.** Artefactual nanoparticle displacement at cryosection surface. **(A, B)**: Images of 40µm-thick gut cryosections from adult zebrafish exposed for 24h to 0.05% red fluorescent nanoparticles. Pictures are maximal intensity projection of 5 confocal pictures taken every two micrometers. Whereas acquisitions in the core of the cryosection reveal low nanoparticle uptake in the lamina propria **(A)**, a larger number of nanoparticles are detected in the same region at the surface the cryosection **(B)**, suggesting that PLA nanoparticles may be displaced during immersion steps of the staining protocol. **(C)**: Epifluorescence image from similar cryosections, cut across a nanoparticle-rich region of the gut lumen. The smear of nanoparticles originating from the lumen suggests that the cryosecting blade may displace nanoparticles at the surface of sections. Lu (Lumen), Lp (Lamina propria), Ep (Epithelium), I (Intestines), P (Pancreas), L (Liver). Scale bar: 10 µm


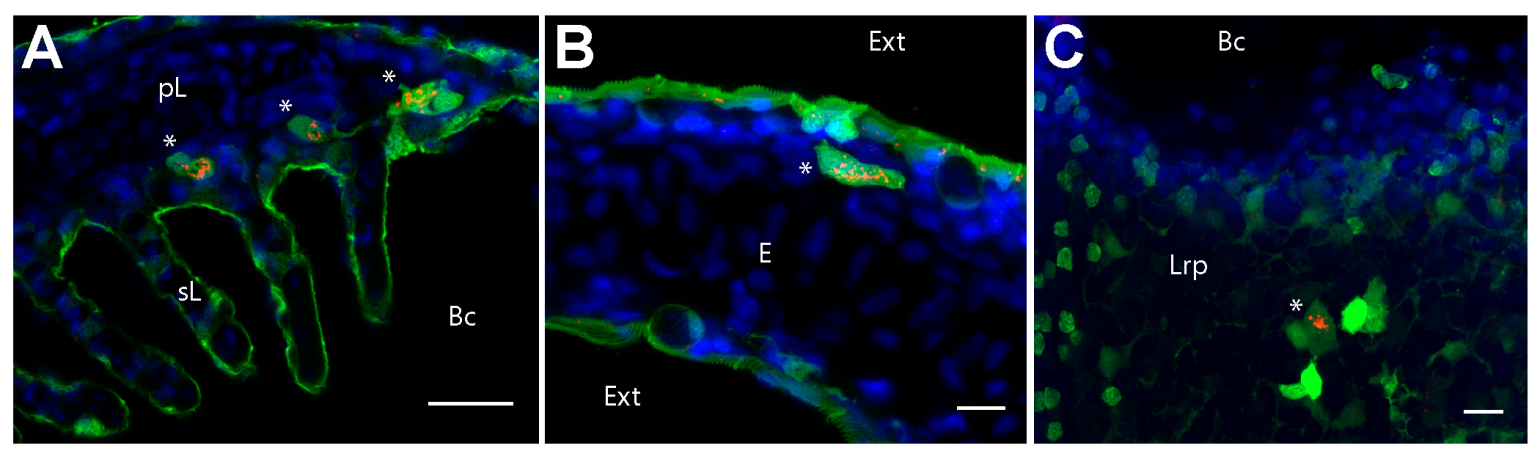


**Supplementary Figure 3.** Cells with high intracellular and granular PNA staining express MHC2. Confocal images of gill **(A)**, skin **(B)** and thymus **(C)** cryosections from mhc2dab:GFP adult fish, following PNA (red) and DAPI (blue) staining. Single optical section **(A)** or maximal intensity projections from 9 **(B)** or 5 **(C)** optical sections taken every micrometer. pL (primary Lamellae), sL (secondary Lamellae), Bc (Branchial cavity), Ext (External environment), E (Epidermis), Lrp (Lympho-reticular parenchyma). Scale bar: 10 µm **(B, C)**, 20 µm **(A)**.
